# Facilitating choice when engaging young people with disabilities: reflections from co-researcher training

**DOI:** 10.1186/s40900-024-00626-7

**Published:** 2024-08-26

**Authors:** Emma Nicholson, Laurel Mimmo, Lauren Christophers, Maria Elena Costa Sa, Trish MacKeogh

**Affiliations:** 1 School of Psychology, Faculty of Health and Science, Dublin City University, Glasnevin Campus, Dublin 9, Ireland; 2 Sydney Children’s Hospital Network, High St, Randwick, NSW 2031 Australia; 3 UCD School of Medicine, University College Dublin, Belfield, Dublin 4, Ireland; 4 Irish Network Against Racism, 28 North Great George’s Street, Dublin 1, Ireland; 5 Central Remedial Clinic, Vernon Ave, Clontarf East, Dublin, D03 R973 Ireland

**Keywords:** Young people; disabilities, Inclusive research, Choice, Patient and public involvement

## Abstract

**Background:**

A collaborative approach is critical in inclusive research and should incorporate taking time to build relationships with co-researchers based on trust and shared understanding. Involvement can often be seen as tokenistic and in order to avoid this, it is important to provide opportunities for people to exercise choice throughout the research process.

**Main body:**

The current paper outlines learnings from a co-researcher training process for young people with disabilities to identify the ways in which meaningful choice can be facilitated with this group. While conducting training of co-researchers in topics such as research methods, we were continuously led by the group with regards to the directions that the sessions took and promoted problem solving with the group to accommodate the unique needs of all members. The overall aim of a wider project was to develop research capacity in a group of young people with disabilities through co-researcher training and this paper will report on learnings from this work with regards to how we sought to provide opportunities for the co-researchers to exercise choice within research projects. Feedback from the group of young people highlighted the variety of needs and expectations that must be accommodated in such a process and therefore, allowing them to dictate the extent and manner of their engagement is key. Young people with disabilities are a heterogeneous group and therefore, some methodologies and ways of working required adaptation in order to facilitate meaningful choice and engagement for all.

**Conclusion:**

Providing meaningful opportunities for demonstrating their choices, in relation to elements of research projects, is a critical component of facilitating a rights-based approach when conducting co-research and requires researchers to cede some level of control over the research process to co-researchers. This can be difficult to achieve in practice and researchers must continuously reflect on their own practice and be willing to change and adapt throughout the process.

**Supplementary Information:**

The online version contains supplementary material available at 10.1186/s40900-024-00626-7.

## Abstract (plain language summary)

It is very important that all members of society can be part of research teams so that researchers can develop projects that will provide good outcomes, however, not all people with disabilities have opportunities to take part in research projects. This project supported a group of young people with disabilities to develop research skills through a set of workshops and this paper describes what the researchers learned from these workshops. This paper will help researchers to understand how best to capture the voices of this population for future research. Young people with disabilities are all different and must be supported to give their opinion in the way that suits them best. Researchers must always think about the ways that they work and change how they work if it is not good for supporting young people with disabilities to work with them on research projects.

**Supplementary Information:**

The online version contains supplementary material available at 10.1186/s40900-024-00626-7.

## Introduction

Inclusive research with people with intellectual disabilities is a form of public and patient involvement (PPI) that can offer benefits both for the quality of research aimed at capturing lived experiences and for the capacity building and empowerment of co-researchers [1]. Researchers with lived experience can be included in advisory roles, whereby they provide feedback on the overall project, leading/controlling roles or as a part of collaborative groups [2]. Some reported benefits of patient engagement within health research include improved enrolment and retention in services [3] while also strengthening their capacity to make good health decisions [4]. For people with disabilities, benefits might include the opportunity to have a voice and visibility in a field that has typically excluded them as well as, for instance, gainful employment in service evaluations of social care. However, a challenge within inclusive research is addressing power imbalances in order to facilitate people with disabilities to exercise choice within a research project.

Within a collaborative approach, time is taken to build relationships with co-researchers based on trust and roles are established for people with disabilities and for others. This might involve a training process where researchers with lived experience learn about the research process in order to prepare them for conducting research in the future. For successful collaboration to occur, it is essential that a solid “architecture of involvement” is created [5] including pre-planning, training of co-researchers and team working, flexibility and problem solving within the team [1]. This helps to ensure co-researchers are not overburdened but have authentic involvement in the project from the start. It is essential that the research team strives for equality, avoids tokenistic involvement, respects the autonomy of researchers and safeguards their dignity [1]. Authentic rather than tokenistic involvement in inclusive research or public patient involvement can be prevalent, with research partners and co-researchers sometimes included at lower levels of consultation rather than active involvement from an early stage of the research [6, 7]. However, there is a growing literature outlining research studies and approaches which demonstrate how co-researchers with disability such as intellectual disabilities can successfully contribute to all elements of a research project [2, 8, 9].

The human rights model of disability emphasises the inherent dignity of people with disabilities and situates people with disabilities at the centre of all decisions affecting them [10]. However, while the human rights model of disability works well within organisation and governmental structures that may have typically excluded people with disabilities, it doesn’t necessarily address the broader societal exclusion and lack of choice experienced by people with disabilities [11]. Inclusive research forms a core tenant of the disability rights movement [9] and indeed, active and informed participation in decision-making processes, including disability and age-appropriate assistance for children, are embedded within the UN Convention for the Rights of Persons with Disabilities [12]. In order avoid tokenistic involvement [1], it is important to provide opportunities for people to engage in choice [13]. Embracing a rights-based approach when conducting co-research requires researchers to cede some level of control over the research process to co-researchers [8]. Negotiating control imbalances in decision-making processes can be contextually driven where control is continuously shifting between the person with disabilities and an external person, which in the context of research is typically academic partners [8, 14]. The challenge in research is attempting to identify approaches that facilitate the ceding of power to individuals who are experts in their own lives.

Young people and children with disabilities experience significant health inequities compared to their peers [15, 16]. They are more likely to experience poor quality care in hospital, to experience adverse events from healthcare than their peers [17–19], and less likely to engage in health promotion and disease prevention activities [20]. People with disabilities experience increased prevalence of chronic conditions [21] have a documented decreased life expectancy [22], and are also more likely to be hospitalised [23]. Overall, it appears that people with disabilities experience a “cascade of disparities” whereby they have higher rates of adverse health conditions, disparities in attention to care needs, disparities in preventative care and health promotion practices and disparities in equitable access to healthcare [24]. Given the extent of these health disparities, it is important that research that is driven by people with disabilities to examine their lived experiences of health and health care. Moreover, methodologies and ways of working which can support them to exercise choice within such research are vital.

The current paper outlines reflections and learning from a co-researcher training process for young people with disabilities in order to identify the most optimal ways to facilitate meaningful choice with this group. The aim of a broader project was to develop research capacity in a group of young people with disabilities by utilising participatory research methods to generate resources to capture their experiences of health and healthcare, and its impact on their lives, which then informed the design of a survey whereby the group collected and analysed data from peers as a means to gain experience doing research. The initial research topic was related to young people with disabilities’ experiences of healthcare; however, we provided space for the group to discuss topics which were most of interest to them. Providing space for the young people to exercise choice throughout the process was a guiding principle for the team and the present paper will share reflections and learnings from this work.

## Method

### Co-researchers

A group of co-researchers (*N* = 8) were recruited to be part of the research team following an information session with the academic researchers which took place in the School. The group comprised of teenagers with intellectual and physical disabilities between the ages of 16–18 years old. Four were females.

### Participating sites

The co-researcher training workshops were held in a school attended by the group from January 2021-March 2021. The numbers of co-researchers and sites were limited to allow the team to develop meaningful relationships which would have been more difficult with multiple groups and sites.

### Ethics

The project was granted ethical approval by the University College Dublin Research Ethics Committee (ref: LS-E-20-204-Nicholson) and Research Ethics Committee (ref number: 72004) in the participating school.

All participants provided informed consent on their own behalf. Co-researchers were provided with easy read information sheets regarding participation by a human rights officer in the School. Two information sessions were held to ensure the co-researchers were fully informed about what participation entailed. After the second session, written informed consent was obtained from both the young people and their parent or guardian. Consent from a parent or guardian was required according to School policy.

### Methods

A co-research methodology with a collaborative approach can capture the lived experiences of this population. Capturing lived experiences of health for people with disabilities through participatory methods could help identify barriers, enablers and mechanisms underlying the identified health inequities for this population [25]. The current study utilised two such methods (1) Photovoice and (2) Body Mapping. It is important to note that the participatory methods were not employed as methods of data collection rather they were adopted for the current workshops to allow for collaborative identification of community issues with co-researchers and to initiate knowledge sharing and/or potential routes for action [26]. The outcomes of these methods then informed a survey which was designed with the co-researchers.

#### Photovoice

Photovoice is a participatory method that was used in the current work to engage the co-researchers on the topic of health, and what it means to be healthy. Photovoice typically involves providing people with a camera to act as recorders and catalysts for change in their own communities and has been used by researchers and co-researchers with various marginalised or seldom heard groups such as rural Chinese women [27], veterans returning to education [28], as well as for examining health issues with people with intellectual disabilities [25, 29]. Photovoice has been established by researchers as a useful participatory method for engaging co-researchers with disabilities in the exploration of health related topics [25, 30], particularly for capturing complex definitions of health. As Photovoice does not rely on verbal communication or cognitive ability, it is accessible for people with diverse communication or cognitive abilities and appropriate for our aims of engaging a group of young people with disabilities in co-research on the topic of health. Previous work examining health beliefs of Latinx people with intellectual disabilities to create appropriate health promotion programmes demonstrated that Photovoice is a feasible method for individuals with disabilities [30]. Further research used Photovoice to engage co-researchers in sharing their experiences of “what it means to be healthy” reported that participants’ complex definitions of health were better captured through this composite of narrative descriptions and visual data than through more traditional research methods [25]. The resulting visual data from this research demonstrated the depth and complexity participants had in their understanding of health. Thus, this participatory method can serve several functions by engaging the co-researchers in the research process, capturing their lived experiences of health and healthcare, and allowing for the collaborative creation of a health-related survey for other young people with disabilities.

The Photovoice method used was adapted to accommodate COVID-19 public health restrictions which were in place at the time the workshops took place and restricted the ability of the group to take photographs beyond a 2-kilometre radius of their home. Thus, rather than merely generating photographs using a camera, the members of the group were asked to select three images they had taken previously or those taken from the internet which they felt adequately represented health and what it meant to them. Each member of the group presented their Photovoice images to the wider group if they wished. They also had the option of presenting just to one member of the research team that they felt most comfortable discussing it with or not at all if that was their choice.

#### Body mapping

Body Mapping is a qualitative participatory method which engages the participants or co-researchers in “storying the self” [31]. This method involves tracing an outline of a person’s body, after which the life-sized outline can be filled in during a creative and reflective process. The produced image can represent multiple aspects of an embodied experience. This method had to be somewhat adapted due to the COVID-19 restrictions in place at the time of the workshops (see Table 1) and was used as a tool for co-researchers to express themselves (thoughts, feelings, experiences as well as likes and dislikes) around the topic of being healthy. The Body Mapping was approached in a sensitive way owing to the recognition that persons with disabilities may have issues around body image or ability to have their shape or outline taken accurately. As with Photovoice, each member of the group presented their body map to the wider group if they wished. They also had the option of presenting just to one member of the research team that they felt most comfortable discussing it with or not at all if that was their choice.

### Workshops

A series of workshops were developed with the aim of building research capacity of young adults with disabilities and investigating different participatory methods to capture experiences which would then inform the design of a survey. The workshops were designed to be mutually beneficial and therefore, the team could test methodologies while the group developed research skills. However, in collaborative discussions between researchers it was agreed that the guiding principle for the entire project should be choice and that the young people would be allowed to lead the process as much as possible. The workshops were planned in advance, however, there was an agreed emphasis on a flexible style to workshop facilitation in which the co-researchers were free to explore different topics and activities as they arose. In line with this, accessible agendas were shared before each workshop (but these were rarely abided by). Table 1 outlines the content of the 5 workshops. In order to promote reciprocity in the group, the research team also contributed to the Photovoice and Body Mapping exercises and presented these to the group.

Where the researchers were unsure of how to approach something (e.g., what participatory methods to use, what to include in a session), the co-researchers were asked what they would prefer. The flexibility and adaptation were also required during the research process as workshops were being developed and scheduled during the COVID-19 pandemic, with public health restrictions changing regularly. This impacted the mode of delivery of the workshops, included in the Table 1. PPI activities in the current study are reported using the GRIPP 2 reporting checklist [32] which can be found as a Supplementary File.

**Table 1:** Outline of workshops

	Planned activities	Changes made
Workshop 1	• Introduction: Perspective taking. “Imagine you had a third eye” activity. • Discussion topic: what is inclusive research and who does it belong to? • Discussion topic: visiting the doctor. • Debrief and reflection.	• Conducted completely online due to Covid-19 public health restrictions. • Co-researchers lead with a different discussion topic – accessibility and discrimination on transport.
Workshop 2	• Introduction: role play – imagine going to buy chocolate but you can’t say the word chocolate. • Photovoice: researchers explain photovoice method by showing their photos. • Body mapping: researchers demonstrate their body maps. • Discussion around which participatory method co-researchers would like to do during these workshops	• Conducted completely online due to Covid-19 public health restrictions. • Co-researchers expressed interest in trying both methods rather than choosing between the two.
Workshop 3	• Introduction: fun activity • Looking at example photos and body maps. • Activity: Do an example body map or example photovoice • “Homework”: photovoice or body mapping on the topic of what does it mean to be healthy?	• Researchers attended virtually, co-researchers attended together in the School due to changing Covid-19 public health restrictions. • Initial plan for the workshop was to present and facilitate either photoshop or body mapping based on group consensus in workshop 2. • As co-researchers were interested in trying both methods, the choice was left up to each individual on whether to use a body map or photovoice on the topic of what it means to be healthy.
Workshop 4	• Introduction: if you were an animal, what kind of animal would you be? • Group sharing their map or photo and discussion. • Reflection: what did you like about photovoice/body mapping? What did you not like?	• Researchers attended virtually, co-researchers attended together in the School due to changing Covid-19 public health restrictions.
Workshop 5	Creating a survey	• Researchers attended virtually, co-researchers attended together in the School due to changing Covid-19 public health restrictions.

## Results

### Feedback from co-researchers

Each member provided oral feedback on their experience to one member of the team to reflect on their experience with the workshops and to note the aspects of the workshops that stood out most clearly for them. Three members of the group enjoyed the discussions where some liked having their opinion heard and others enjoyed learning what the team thought about different topics. On balance, another member did enjoy the discussions that took place as part of the workshops but did not enjoy conducting the survey.

“I loved chatting […]. It was nice to be asked my opinion. Like my actual opinion. I didn’t like the surveys as much”.[Student, 1]

Other members of the group enjoyed the survey element and found that it was relevant to other parts of their life.

“Sometimes I found the talking all a bit much or I would get fed up if I wasn’t talking. I liked doing our own surveys. I remembered a lot this year when we covered surveys again. That made it easier”. [Student, 2]

“I really enjoyed chatting and having fun with it. It kind of got me thinking about different things and topics. Making the surveys helped me come up with some good ideas for Maths […]. It was nice getting to understand what other people thought about things”. [Student, 3]

“I liked chatting with my friends and going around to different classes for the survey.” [Student 4].

COVID-19 public health restrictions in place at the time of the workshops necessitated the use of the remote environment to conduct sessions which worked well for some but not others. One group member, who would not be comfortable speaking in a group, preferred the remote environment as it allowed them to participate and give their opinion by typing into the chat function.

“I found the discussions hard at times when in class. I found the Zoom easier because I could think and type it out without worrying. It was easier to get someone else to say how I felt. I liked that I could be included”. [Student, 5]

On balance, another student, for whom English was not their first language, felt that they struggled with English in the remote environment which caused them to disengage from the session.

“I found the English too hard on [the online environment]. I wasn’t that interested”.[Student, 6]

With regards to support, one member of the group had a sibling support them during some of the sessions and they felt that this allowed them to get their opinion across as the sibling knew them so well.

“I enjoyed the sessions most when I was with my sister Sarah^1^ at home. I found it easier to take part because Sarah knows me so well and could help me communicate how I felt. She understands how hard it can be for me sometimes”. [Student 7]

### Field notes and reflections

#### Working with each person on their own terms

Every person who participated in the workshops had different preferences and requirements for support in order to facilitate their desired degree of participation in the workshops. For instance, some members of the group had support from another person and there were no limitations on who could provide this support. As discussed above, for one member, a sibling provided this support and was an invaluable asset to them because they were non-verbal and the sibling was well placed to interpret their needs and views. For others, a Special Needs Assistant (SNA) who they worked with in their classroom setting provided the support. The support person had differing roles depending on their relationship with the person. Some provided a sounding board for the person and allowed them to discuss ideas before they were shared with the group, if the team member had communication challenges, they sometimes relayed the views of the person to the group. Given the remote nature of many sessions, the presence of a support was not specified before the session but it was important that it was facilitated as the workshop progressed.

We also found that we needed to be very flexible with attendance and participation. Scheduling is an unavoidable requirement, however, in some cases, a member of the team may not have wanted to take part on a given day and therefore, the session proceeded with those who wished to attend. Moreover, there was some work to be done outside of the groups and we were mindful that this was being done alongside other commitments. There was never an obligation to take part in all workshops or all activities and it was entirely driven by each team member. Regarding engagement, we made the group aware that they didn’t have to share everything with the wider group but that if they wanted to share something individually or if they thought of anything outside of a workshop that could speak to any team member. Some members did speak outside of the workshop but only to team members who they saw on a regular basis as part of their daily routine. Establishing accessible means for the group to provide feedback in a way that works best for them would be of benefit for future work.

### Maximising the remote environment

The workshops were scheduled to take place during the implementation of public health restriction necessitated by the COVID-19 pandemic. The early workshops took place entirely online while the later workshops were a hybrid where the co-researchers were in a room with two members of the team and three members joined remotely. While this was not originally intended, there were pros and cons to this approach that will guide future participation work. Some team members flourished in the remote environment while others struggled and this was reflected in the feedback. For instance, the ability to share their views and opinions through the text chat functions on remote platforms was a great support where they would not be willing to speak in a group. On balance, for one team member without English as a first language, it was difficult to understand online. For some, the nature of the environment appeared to have little bearing on their level of participation.

### Setting the tone of a session to foster a relaxed atmosphere

While some team members were familiar with each other, others were not and therefore, taking time to establish relationships between the team was an invaluable component of the workshops. Relationships will naturally form over time and a number of simple exercises carried out in the workshops helped to facilitate this within the short timeframe available in each session. For instance, at the beginning of each session we held an ‘ice-breaker’ which was a short and light-hearted exercise that each member was given the option of taking part in and which also included the researchers on the team. These exercises often generated many laughs from the group and helped to foster a relaxed atmosphere which we hoped would make the group more comfortable in sharing their views and opinions. It was important that the researchers on the team also participated in this. We found these exercises to be very effective and while they often took more time than initially intended, it was worth the time they took.

### Flexibility with methods and content of workshops

Ahead of each workshop, the team had prepared accessible agendas and PowerPoint slides to guide each workshop. While the slides were of benefit when we were trying to introduce some of the concepts and activities such as photovoice and body mapping, the agendas were rarely adhered to in full. It became apparent very quickly that attempting to dictate a session like a traditional research meeting was detrimental to facilitating choice. In order to attempt to conduct the sessions on the co-researchers’ terms as much as possible, we made an effort to be led by the group in terms of the direction the workshops took and the activities and discussions that took place.

Frequently, the sessions took a turn that was not anticipated. For instance, when we were discussing the purpose of participation in research and why it is important that their voices are heard, it launched an in-depth discussion about how people with disabilities experience inequality in society in areas such as transportation. This resulted in other parts of the session being dropped but it was a truly valuable exercise for the group with regards to becoming comfortable expressing their opinions that it was important not to stifle the discussion. In another instance, when discussing what health meant to them, they spoke a lot about the importance of mental health and how happiness was a key part of health. Some of the richest information emerged from the unprompted moments and it is important to allow these to happen organically and not to stifle discussion in order to follow an agenda.

Public health restrictions in place due to the COVID-19 pandemic also required flexibility in terms of how we operationalised some of the methodologies used. For instance, traditionally Photovoice would involve individuals taking photographs, however, the public health restrictions placed limits of the distance people were allowed to travel from their homes and self-isolation guidelines meant some may have been isolating at other times. As a result, we encouraged the group to use any existing photograph’s that they may have had on their devices or to download photographs from the internet in place of taking photographs. Moreover, the approach of selecting images was a unifying experience for the group as the same method was used for everyone regardless of the ability of the participant.

### Limitations

One key limitation of this paper is that the group of young people with disabilities did not contribute to the write up of the work nor did they provide evaluations of our work. With regards to the write up, this was primarily a practical consideration as they had moved on from the school where the workshops had been held and it was not possible to contact them in the year after the workshops took place. For the evaluation of our work, we did not have ethical approval to conduct this type of work. As a result, the present paper is primarily presented from the researcher’s perspective. Moreover, while efforts were made to ensure that a broad spectrum of young people were able to be included in the team, the group self-selected to take part and therefore, those who may be predisposed to take part in such work were potentially over-represented.

### Lessons learned and recommendations

- **Do not be overly prescriptive about the process**: Preparation and planning are critical but it is important to be flexible during a session as facilitating choice necessitates being led by the co-researchers and will lead to more meaningful engagement. This may require adapting an established methodology, but it is vital in order to ensure more people can engage in a way that works for them. The solution to such challenges are provided by the people themselves so trust the person with disabilities to create their own solutions.
- **Bespoke and responsive arrangements**: Remote meetings can be very beneficial. Provide choice for groups and be prepared to change on a session-by-session basis. Some remote and some in person can work with the right set up. Supporting different types of engagement and choice with regards to means of participation . Bespoke and changing arrangements may be required.
- **Take time to establish relationships**: It is important that researchers spend more time building links and trust in the community and engaging in participatory research. The nature of funding and research does not often support this process but it is integral to facilitating choice. Projects are often time sensitive, however, ice-breakers at the beginning of a session can support this in a short-time frame. Reciprocity is important for relationships so the research team should also participate and share their experiences too.
- **Facilitate ongoing feedback**: Allow for co-researchers to input and provide feedback outside of the specified time frame in a session or workshop which is accessible with the potential for anonymity. We found this challenging but using online spaces such as Jam Boards or providing a physical space where they can leave written feedback may be useful. The co-researchers may tell you their preferences however, it might be necessary to provide some options to them.
- **Understand co-researchers’ motivations and goals**: It is important to recognise that co-researchers will have different reasons for participating in research that may go beyond the final goals and deliverables of a given project, and these may vary as a project progresses. Taking time to understand these motivations will help a research team ensure that co-researchers are meeting their chosen goals.

## Conclusion

The UNCRPD states that decision-making processes must include active and informed participation of people with disabilities [10] and the research process is no exception. Providing meaningful opportunities for choice [13] is a critical component of facilitating a rights-based approach when conducting co-research and requires researchers to cede some level of control over the research process to co-researchers. This can be difficult to achieve in practice and researchers must continuously reflect on their own practice and be willing to change and adapt throughout the process. Young people with disabilities are a heterogeneous group and therefore, some methodologies and ways of working will need to be adapted in order to facilitate meaningful choice and engagement for all. Moreover, it is critical that opportunities for involvement are not hampered by gatekeepers which restrict participation of groups deemed vulnerable when actually limiting their ability to fully engage in society. Research is an evidence base for policy change and those who are most affected should be supported to become architects of that change.

### Electronic supplementary material

Below is the link to the electronic supplementary material.

Supplementary Material 1

## Data Availability

This paper does not report on original data.

## Footnotes

1 Names have been changed.
